# ﻿More discussion of minimalist species descriptions and clarifying some misconceptions contained in Meier et al. 2021

**DOI:** 10.3897/zookeys.1110.85491

**Published:** 2022-07-05

**Authors:** Michael J. Sharkey, Erika M. Tucker, Austin Baker, M. Alex Smith, Sujeevan Ratnasingham, Ramya Manjunath, Paul Hebert, Winnie Hallwachs, Daniel Janzen

**Affiliations:** 1 The Hymenoptera Institute, 516 Saguache Dr., Florissant, CO, 80816, USA; 2 Biodiversity Outreach Network; South Lyon, Michigan, USA; 3 Department of Biological Sciences and Center for Biodiversity Research, University of Memphis, Memphis, Tennessee, USA; 4 Department of Integrative Biology, University of Guelph, Guelph, Ontario, Canada; 5 Centre for Biodiversity Genomics, University of Guelph, Guelph, Ontario, Canada; 6 Department of Biology, University of Pennsylvania, Philadelphia, PA 19104-6018, USA

**Keywords:** Barcode Index Numbers (BINs), biodiversity, Braconidae, consensus barcodes, DNA barcodes, Ichneumonoidea, taxonomic impediment

## Abstract

This is a response to a preprint version of “A re-analysis of the data in Sharkey et al.’s (2021) minimalist revision reveals that BINs do not deserve names, but BOLD Systems needs a stronger commitment to open science”, https://www.biorxiv.org/content/10.1101/2021.04.28.441626v2. Meier et al. strongly criticized Sharkey et al.’s publication in which 403 new species were deliberately minimally described, based primarily on COI barcode sequence data. Here we respond to these criticisms. The following points are made: 1) Sharkey et al. did not equate BINs with species, as demonstrated in several examples in which multiple species were found to be in single BINs. 2) We reiterate that BINs were used as a preliminary sorting tool, just as preliminary morphological identification commonly sorts specimens based on color and size into unit trays; despite BINs and species concepts matching well over 90% of species, this matching does not equate to equality. 3) Consensus barcodes were used only to provide a diagnosis to conform to the rules of the International Code of Zoological Nomenclature just as consensus morphological diagnoses are. The barcode of a holotype is definitive and simply part of its cellular morphology. 4) Minimalist revisions will facilitate and accelerate future taxonomic research, not hinder it. 5) We refute the claim that the BOLD sequences of *Plesiocoelusvanachterbergi* are pseudogenes and demonstrate that they simply represent a frameshift mutation. 6) We reassert our observation that morphological evidence alone is insufficient to recognize species within species-rich higher taxa and that its usefulness lies in character states that are congruent with molecular data. 7) We show that in the cases in which COI barcodes code for the same amino acids in different putative species, data from morphology, host specificity, and other ecological traits reaffirm their utility as indicators of genetically distinct lineages.

## ﻿Introduction

The world is in crisis. Not only are we facing massive global species extinctions, primarily of species yet to be discovered, but every year we lose more and more of the taxonomic expertise needed to describe and record these species before they are gone. While neither the ongoing extreme loss of biodiversity (Cardinale et al. 2012; IPBES 2019) nor the loss of taxonomic expertise is questionable (Wheeler 2014), a satisfactory and viable solution has yet to be found.

To help address the taxonomic impediment and to address some of the many issues taxonomists struggle with in their efforts to describe hyper-diverse fauna before species are lost, Meierotto et al. (2019) proposed a new method to describe species more quickly, to give them handles with which all of humanity can diagnose and treat them. This method primarily uses COI barcodes as indicators of species, largely leaving time-consuming, and at times more ambiguous, morphological description up to future work if desired or needed. This protocol was later named the “Minimalist Technique” by Sharkey et al. (2021a) who employed it to describe 403 new species of Costa Rican braconids. All studies using the Minimalist Technique thus far have focused on taxa within the hyper-diverse hymenopteran superfamily Ichneumonoidea, a taxon in which COI barcoding has already been established as useful and often essential in delimiting species boundaries. As one may imagine, this primarily molecular-based approach has inspired intense debate within the taxonomic community, a community that traditionally regards morphological descriptive work as essential for species description. Not only has the value of the Minimalist Technique been debated, but the appropriateness of its use has been argued, despite having originally been proposed to try to address the taxonomic and biodiversity crises.

Meierotto et al.’s (2019) paper was quickly followed by criticisms from Zamani et al. (2020). This was then followed by the Sharkey et al. (2021b) reply addressing and refuting the points brought up by Zamani et al. (2020), and then by Sharkey et al.’s (2021a) paper publishing 403 new species using the Minimalist Technique. Sharkey et al.’s (2021a) paper was followed by a detailed response from Meier et al. (2021) strongly criticizing not just the method used to evaluate the molecular data used in both Sharkey et al. (2021a) and Meierotto et al. (2019), but in using BINs (Barcode Index Numbers) and Barcode of Life Data System (BOLD) services in general. Subsequent discussion recently published by Zamani et al. (2022) offers support of Meier et al.’s criticisms, yet neither paper offers a realistic alternative solution to the problems faced by the taxonomic community and its relation to the greater global populations on which it depends.

Here, we attempt to constructively continue the ongoing discussion. Despite the harsh criticism and lack of alternative solutions, we appreciate the considerable amount of time Meier et al. (2021) put into their critique and will address some of the points they brought up. We respond here to a preprint version of Meier et al. (2021). There are several other preprint versions available, and some of these can be found at https://doi.org/10.1101/2021.04.28.441626. The article was recently published in Cladistics (Meier et al. 2022) but our manuscript was too far along to incorporate the edits contained there. We also reiterate the value the Minimalist Technique has for many taxonomists, particularly those working in Ichneumonoidea and other diverse taxa; of course, like any other tool, it may not work for every taxonomist in every situation.

## ﻿Discussion

### ﻿Revision methods

Towards the end of their paper, Meier et al. (2021) stated, “Overall, we must conclude that the methods used in the revision are too poorly described to fully understand how the authors gathered and treated evidence.” Yet, in their own paper, Meier et al. stated that Sharkey et al. (2021a) equated species with BINs. As the methods described in Sharkey et al. (2021a) clearly explained, the use of BINs in the Minimalist Technique is the first step in grouping together what are likely to be conspecific specimens. This is analogous to when physical specimens are sorted to morphospecies into unit trays in museum drawers, based on color, morphology, and size, before further inspection, when they may or may not be found to be conspecifics. The BIN is effectively a DNA-based unit tray. Subsequent to BIN sorting, morphology, NJ tree topology, sequence length, and often host and other ecological data, were considered to produce each species boundary in Sharkey et al. (2021a).

### ﻿Consensus barcodes

Meier et al. (2021) spent a considerable amount of effort criticizing the consensus barcode approach to diagnosing species. It should be noted that the consensus barcode was not mentioned in the description of the Minimalist Technique (Meierotto et al. 2019). It was included in later papers (Sharkey et al. 2021a, 2021c) only to satisfy the rules of the International Code of Zoological Nomenclature (ICZN), just as consensus morphological character states are used for the same purpose. Consensus barcodes are simply an average of all the barcodes that belong to a species, just as “foretibia 0.02–0.04 mm” is part of a morphological diagnostic. It is exactly analogous to species-level descriptions/diagnoses of colors patterns, which are famous for slight, and difficult to decipher variation. For example, if a species is polymorphic at a particular site, an International Union of Pure and Applied Chemistry (IUPAC) ambiguity code is used to indicate the polymorphism. The program employed to generate consensus barcodes can be found at https://www.phorid.net/DNAbarcode, and information on how it functions to produce a consensus barcode can be found here https://www.phorid.net/DNAbarcode/about_conSeq.php. The Sharkey et al. (2021a) revision was meant to describe species that were reared, and/or Malaise-trapped, in Costa Rica as part of Dan Janzen and Winnie Hallwachs’s massive caterpillar and parasitoid rearing program. The barcode clusters generated, closely matching BINs, often contained specimens already on BOLD that the authors did not examine. This is the same as in the cases of many morphologically described species encountered in this or that museum, that the authors of the species never see. As mentioned, the consensus barcode is there only to satisfy the code, but it does represent the species as much as any consensus morphological trait, and the barcode of the holotype certainly falls within the BIN that contributes the consensus barcode. Much more collecting, barcoding, and examining of specimens from Costa Rica and additional locations will achieve more robust concepts, but no more so than for any classical morphological approach.

Rather than using consensus barcodes as the tool to identify a species, Sharkey et al. (2021a) and Meierotto et al. (2019) presented an example of how species identifications could proceed using COI barcode data. One of the primary aims for introducing the Minimalist Technique was to create the ability for anyone to quickly identify a specimen on BOLD after acquiring a COI barcode from the specimen (if a similar sequence exists in BOLD already). Whenever a sequence is submitted to BOLD the user is given an identification for that sequence. The level of the identification can vary depending on the length of the sequence submitted, but at a minimum it usually allows the user some degree of confidence that they sequenced the correct specimen and did not amplify the barcode of a contaminant. When something as precise as a species-level identification is returned, best practice is, of course, for users to double-check the quality of the determination. Users can do this by going through many of the same steps that Sharkey et al. (2021a) and Meierotto et al. (2019) went through to name species, e.g., checking nearest neighbors, images, and locality data. Perhaps the most effective and efficient method is to generate a simple neighbor-joining (NJ) tree on BOLD that includes the submitted sequence; this is an option given on BOLD. The user can then see how distant the submitted sequence is from a reliably identified specimen, preferably the holotype if it has been sequenced. The distance from one specimen’s sequence to another, with a check of any similar pre-existing specimen image, will determine the degree of confidence the user may have.

The BIN code, in and of itself, is only meant as a communication and organization handle. This is not only because BINs do not equal species, but also because in rare cases the codes for BINs may change over time. One BIN could split into several BINs or be subsumed into another BIN and completely disappear. This happens, though rarely, when two narrowly separated BINs converge due to increased specimen sampling. One of the two BINs takes precedence, and the other is lost. This is especially true for under-sampled taxa, and that is the case for almost all species of hyper-diverse taxa such as most tropical Ichneumonoidea. Neither the BIN code nor the consensus sequence has any relationship with the new species described by Sharkey et al. (2021a) other than to give the specimens a preliminary sort and to satisfy the ICZN rules. While it would have been ideal to have better explained the use of BINs and consensus barcodes in the methods section of the original paper, the assumption during preparation was that it would be self-evident. As that was clearly a poor assumption herein we strive to correct our error.

### ﻿Research depth

Meier et al. (2021) criticized the Minimalist Technique and Sharkey et al. (2021a) for leaving the heavy taxonomic work for future revisors and suggested that the authors should have done more in-depth research themselves. Unfortunately, Meier et al. (2021 took part of the paper out of context and thus misunderstood what was written. Meier et al. wrote, “The authors [referring to Sharkey et al. 2021a] stated: “… we view barcode-based descriptions as a first pass in an iterative approach to solve the taxonomic impediment of megadiverse and under-taxonomically resourced groups that standard technical and biopolitical approaches have not been able to tackle. This means that Sharkey et al. delegate the critical ‘iterative’ work to future generations of taxonomists. They will have to start revisions by first revisiting the species descriptions, types, and specimens of Sharkey et al.’s species to resolve species boundaries based on data that should have been collected and analyzed at the time of description.”

To provide proper context, the following is the entirety of what was written in Sharkey et al. (2021a) that was unnecessarily criticized, “As we have made clear in the Meierotto et al. (2019) paper and here, we are not proposing that comprehensive revisions that include keys and morphological diagnoses be abandoned. Rather, we view barcode-based descriptions as a first pass in an iterative approach to solve the taxonomic impediment of megadiverse and under-taxonomically resourced groups that standard technical and biopolitical approaches have not been able to tackle. For example, if a taxonomist wishes to integrate these elements [morphological keys etc.] to *Zelomorpha* or any of the taxa that we review below they will have a great starting point (and resources). When a large number of specimens, from a wide geographic range, are barcoded, effective morphological keys may be written, and old museum specimens will regain their value to go along with their barcodes.”

The Minimalist Technique first-pass approach does not preclude the addition of morphological data. If a future researcher wishes, for example, to write a key to the species of *Zelomorpha* of Costa Rica, they have a head start, with the species well-defined by sequences, images, and often host data. The entire reason this approach is termed “a first pass” is to suggest that it will not be perfect and that more collecting, and more barcodes will refine the species limits. Meier et al.’s suggestion that we should have collected and analyzed more data at the time of description seems naïve. The entire point of the approach used by Sharkey et al. (2021a) is to speed up the description process. If more data means more specimen collection, more sequence generation, and the addition of more morphological data, then the purpose is defeated, and in the case of hyper-diverse taxa there is no practical end point. It should be noted, however, for those unfamiliar with taxonomic processes, that anyone working on a deep revision of a taxonomic group, particularly those that include keys or new descriptions, should always revisit previous species descriptions, types, and other information previously documented. Therefore, taxonomists needing to work with species as described by Sharkey et al. (2021a) are starting at a huge advantage with the availability of sequences, images, often host information, and known type specimen locations.

Despite the clear explanation given in Sharkey et al. (2021a) on how beneficial the Minimalist Technique can be for producing more descriptive future works, we do not believe generating these products now to be a good use of resources. It is doubtful that writing keys and extensive morphological descriptions for many hyper-diverse groups is of value until there is a taxon-specific need. This is due to the fact that the keys to most species-rich taxa do not usually work, and likely never will. Likewise, the morphological descriptions are often ineffective in confirming species identity. They are rarely, if ever, used, despite the extensive amount of time required to produce them. The pointlessness of such keys is described in detail by Sharkey et al. (2021c). The high species-richness of these enormous genera make it unreasonable to imagine that at this point in our sampling all of the species and their potential discriminatory traits are adequately sampled and understood.

### ﻿BIN stability

Meier et al. (2021) made the statement that BINs are unstable. This is not news to us and is why we do not simply name BINs. To demonstrate instability, Meier et al. compared the BINs supported by the BOLD algorithm with three other methods to group species. They then showed that as the size of the data sets increased, fewer and fewer BINs agreed with the other three methods of species delimitation. This is not at all surprising and is actually the expected result of the exercise. If more than three other methods were used to delimit species, the overlap would decrease even more. That is the nature of being different. We reiterate here that BINs were used as a preliminary sorting tool, just as a preliminary morphological approach might sort specimens by color and size. Interestingly, Meier and others investigated the species of Swedish Phoridae (Diptera) and concluded that the BIN algorithm was more efficient than the other three methods in delimiting species boundaries and that it matched 85% of their species concepts that were the result of morphological and COI-based data (Hartop et al. 2021). We also used morphology, but our concordance with the BIN sorts was well over 90%. This is not an unusually high number for the Braconidae. There are examples of conventional braconid revisions in which BIN concordance with species concepts is 100%, e.g., *Glyptapanteles* (Arias-Penna et al. 2019) with 136 species, and *Prasmodon* (Fernandez-Triana et al. 2014) with 16 species. Both of these publications include morphological keys and descriptions. BINs vary in their effectiveness from taxon to taxon, and for a very few groups of organisms, BINs may not work at all. Author DHJ, who has spent thousands of hours reading multi-thousand-member NJ trees pre- and post-BIN application, has found that BINs are vastly more expeditious than any other kind of specimen sorting for later taxonomic study. For example, try to imagine the difficulty of adding 30 new species morphologically into an ichneumonid pool of 30,000 specimens representing at least 2,000 species, and their intraspecific variation, without using BINs.

In their figure 3 Meier et al. showed that 15 specimens that are currently in BOLD:ABY5286 (*Chelonusscottmilleri*) bounced around in six other BINs before settling in the current BIN in July 2013. Although it is appropriate to analyze BINs in the manner used by Meier et al., their utilization of BIN historical assignments from 2009 to 2013 is not. Assignment data is available in the BOLD audit trail from 2009, however it is only relevant beyond July 2013, when Plos One accepted the BIN publication (Ratnasingham and Hebert 2013). Before this publication, the system was in a state of flux. For example, assignments prior to 2011 were based on Single Linkage Clustering alone, and the RESL algorithm was deployed in 2012. Parameter and algorithmic optimization took place throughout this period. The BIN system was developed directly on the BOLD platform for only one reason: to gain feedback from users with taxonomic expertise. During this time, there was no expectation of BIN persistence, and the BIN database was reset multiple times. This methodological oversite in Meier et al.’s analysis results in the inflation of BIN splits, merges, mixtures, and deletions, all of which make little sense when reviewing actual sequence divergences.

As a final note on this topic, we point out that, like BINs, species names are also unstable, with concepts often changing substantially over time and names subsumed by synonymy and changed with homonymy. Meier (2016) himself demonstrated this using the example of *Drosophilamelanogaster*.

### BOLD Systems data quality

Meier et al. (2021 made several criticisms on the quality of the data on BOLD. The first of these concerns a set of barcodes in one of Sharkey et al.’s (2021a) species. Meier et al. stated, “BOLD:ABX6701 was described by Sharkey et al. (2021a) as *Plesiocoelusvanachterbergi*. The consensus barcode includes two single indels and was presumably obtained from the seven “barcode-compliant” sequences in BOLD Systems (as of April 18, 2021). Only one of the seven barcodes is translatable to amino acids with the remaining six having deletions. Sequences showing these attributes are often derived from pseudogenes, and it is conceivable that *P.vanachterbergi* was described based on paralogs. Note that this is likely due to a lapse in quality control because BOLD is supposed to check for shifts in reading frames (Ratnasingham and Hebert 2013).”

Contrary to the claim that the BOLD sequences of *P.vanachterbergi* are pseudogenes, they simply represent a frameshift mutation. Frameshift mutations in mitochondrial DNA have been uncovered in multiple taxa since the late 1990s (e.g., the ostrich: Harlid et al. 1997; Mindell et al. 1998). Single base-pair deletions are known characteristics for members of the braconid subfamily Agathidinae (e.g., Hrcek et al. 2011; Quicke et al. 2012; Hansson et al. 2015). While frameshifts (+1 or −1) are rare, they appear to be phylogenetically widespread as known examples include diatoms (Oudot-Le Secq and Green 2011), mollusks (Milbury and Gaffney 2005; Capt et al. 2020, ants (Beckenbach et al. 2005), turtles (Russell and Beckenbach 2008), birds (Harlid et al. 1997; Mindell et al. 1998), and parasitic wasps (as addressed in this paper). In the case of Agathidinae, we have consistently recovered the −1 frameshift in taxa from widely divergent localities, from species characterized by host-specific ecologies, and by using multiple primers in different combinations. For mitochondrial DNA sequences characterized by deletions, character removal from the frame can be a sign of pseudogene amplification; however, there are aspects of sequence composition that make this unlikely. The nucleotide composition in sequences with the 1 bp deletions remains highly AT biased, and those substitutions which do appear between species remain biased towards 3^rd^ positions. In addition, the interspecific clusters formed by Agathidinae mtDNA align closely with their host-species biology and identity. Each of these three observations suggests that the sequences are functional rather than pseudogenes. If we were to conclude that the Agathidinae COI sequences on BOLD were pseudogenes, they would be both old (as they are widely geographically and taxonomically dispersed) and young (as they are host-specific), and odd in that they have retained the compositional bias (AT) patterns of substitution that characterize functional gene fragments. Thus, we have worked for over a decade under the more parsimonious hypothesis that the Agathidinae COI sequences are functional and characterized by a novel −1 frameshift mutation that may be cleaved in an unknown bit of RNA editing and that are a characteristic of this subfamily. It appears that Meier et al. (2021) downloaded and aligned COI sequences from BOLD for the BIN BOLD:ABX6701 without consideration for the frameshifts which characterize the subfamily.

Meier et al. (2021) did reveal a clear error “the consensus barcode for *Pseudorhysipolismailyngonzalezae* contains 104 indels, which may be related to the fact that the BOLD fasta download for this BIN consists of data for multiple genes (COI, 28S, 16S, Ef1a). The correct consensus barcode for this species is TGTTTTGTATTTTATTTTTGGTATATGAGCTGGAATAGTTGGTTTATCTATAAGATTAATTATTCGATTAGAATTAGGGGTATCTGGAAGATTATTAGGGAATGATCAAATTTATAATACTATTGTTACATCTCATGCTTTTGTAATAATTTTTTTTATAGTTATACCTATTATATTAGGAGGATTTGGTAATTGATTAATTCCTTTAATATTAGGGGCTCCTGATATAGCATTTCCTCGAATAAATAATATAAGATTTTGATTATTGATTCCATCATTAATTTTATTATTTTTAAGTAGATCAATAAATTTAGGAGCTGGAACGGGGTGAACTATATATCCTCCTTTATCTTCAAGAATTGGTCATAGAGGAATATCTGTTGATTTAACAATTTTTTCTTTACATTTAGCTGGTTGTTCTTCTATTATAGGATCAATTAATTTTATTTGTACAATTTTTAATATAAAAATTAATTTTTTAAAAATAGAACAATTAAGTTTATTTGTTTGGTCAGTTTTAATTACAACAATTTTATTATTATTATCTTTACCAGTTTTAGCTGGTGCTATTACTATATTATTAACAGATCGTAATTTAAATACATCTTTTTTTGATTTTTCAGGTGGTGGTGATCCAATTTTATTTCAACATTTATTT.

Though as discussed, its value is limited, and the hybrid consensus sequence is also diagnostic for the species.

### ﻿Taxon identification

Meier et al. (2021) wrote, “Depending on the time of BIN description, two of Sharkey et al.’s (2021a) wasp taxa would have been described as a fly or stonefly species, respectively” The explanation for this criticism is obviously contamination (Pentinsaari et al. 2020). Author MJS has morphologically sorted through thousands of Hymenoptera records on BOLD to identify entries to the lowest possible level and has previously flagged contaminants. Many others, including staff at BOLD have done the same. Nonetheless, a few contaminants are always possible and, just as using GenBank or in any other taxonomic endeavor, the revisor must check data quality. We note that Meier et al. (2021) did not find any flies and stoneflies in Sharkey et al.’s (2021a) treatment.

### ﻿Morphology

Meier et al. (2021) confused comments made concerning the use of morphology by Sharkey et al. (2021a). Meier et al. wrote, “On the one hand, Sharkey et al. (2021a) argue that morphological evidence is not suitable for braconid taxonomy. On the other hand, they point out that subtle morphological differences often agree with barcode clusters, which would imply that the morphological evidence was misinterpreted.” Our point was that morphological evidence alone is not sufficient to resolve species limits in many taxa. This is clearly the case in the morphological treatments of species of *Alabagrus* by Leathers and Sharkey (2003), as illuminated by Sharkey et al. (2021c). The problem is not that morphological evidence is non-existent, but rather that it is cryptic. There are multitudes of morphological characters, each of which may indicate different groupings of specimens, with no consensus. When COI barcode data are included, there are often one or more morphological characters that are congruent with them. It is more parsimonious to conclude that the morphological characters and COI sequences are congruent due to shared ancestry rather than random chance.

### BINs vs species

Meier et al. (2021) demonstrated that in 16 publications employing barcodes as part of their evidence for determining species limits the species concepts and BINs did not always match, and wrote “We find that, for example, the barcodes for the 10 species in *Astraptes* belong to 5 BINs (Hebert et al. 2004)”. This mismatch is simply because, as in the Sharkey et al. (2021a) publication, authors of these papers did not rely solely on BINs but rather they used larval morphology, microgeographic ecology, and food plants by authors DHJ and WH to determine species based on barcode clusters years before BINs were even a concept; in retrospect they were found to be perfect containers (6 species in one BIN, BOLD:ACE8393 with six obvious shallow-separated clusters on the NJ tree). That BINs do not equal species has been evident to everyone using COI barcodes and was even presented as a characteristic of the BIN system at its inception (Ratnasingham and Hebert 2013). MJS, DHJ and WH routinely sort by BINs, then inspect NJ trees with1–2 thousand specimens, watching for small clusters within BINs that match with other ecological traits. In this fashion they encounter many cryptic species within “well-known” species names (e.g., Hebert et al. 2004).

### ﻿Amino acids

**Figure 1.**
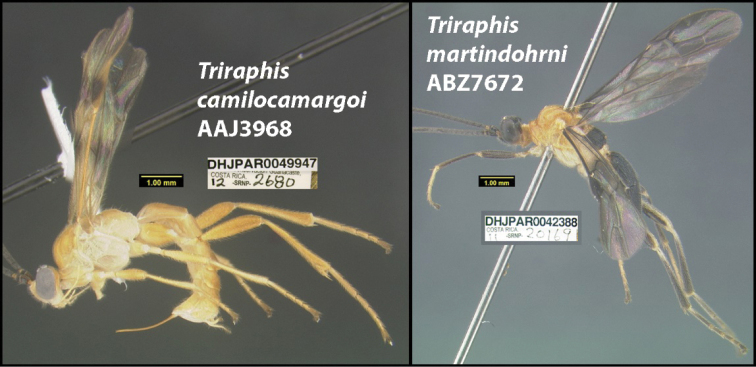
Two species with COI barcodes coding for identical amino acids. Specimen on the left is *Triraphiscamilocamargoi* (BIN BOLD:AAJ3968); specimen on the right is *T.martindohrni* (BIN BOLD:ABZ7672).

Meier et al. (2021) went to great lengths to show that there are 11 cases in which different species named by Sharkey et al. (2021a) had COI sequences that coded for the same amino acids. Meier et al. stated, “Given that most biologists associate speciation with the origin of biologically meaningful differences, describing such BINs as species rests on the hope that the correlation between time of divergence and the origin of new species is strong enough that biologically meaningful differences will later be found.” It is true that Sharkey et al. (2021a) did not check if COI sequences coded for identical amino acids across species, but they did check morphology and host data, and an inspection of these 11 cases shows that the species in each group are distinct morphologically and in all but one case they have non-overlapping host ranges. Contrary to Meier et al., “biologically meaningful differences” are included in the Sharkey et al. (2021a) paper in the form of host data, and images that clearly show morphological differences between species with identical amino acids. One such example are the two species, *Triraphiscamilocamargoi* (BIN BOLD:AAJ3968) and *T.martindohrni* (BIN BOLD:ABZ7672) (Fig. 1). The barcodes of these two species code for the same amino acids, yet, as the images in figure 1 demonstrate they are radically different morphologically, and they have quite different barcodes, which are the code combinations of relevance for all of this discussion, rather than amino acids. What is “biologically meaningful” is a quite different question from taxonomy. An enormous number of traits used in morphological-based taxonomy are of unknown “biological meaningfulness” and barcodes were deliberately chosen for their seeming freedom from natural selection for “use”. Authors DHJ and WH became adherents of the use of barcodes and then BINs, as taxonomic tools in 2004 when it became obvious that for their inventory of very large numbers of species of undescribed tropical Lepidoptera, Hymenoptera, and Diptera, they were a major solution to the taxonomic impediment simply because they matched long established tropical named species. Barcodes and BINs also opened their eyes to other species hiding among the morphologically described species (e.g., Smith et al. 2006, 2007, 2008; Burns et al. 2008; Janzen et al. 2009, 2017; Janzen and Hallwachs 2016). This has continued until today, and the barcode libraries themselves will be major tools for everyone, in any society, rather than just where they currently reside.

## ﻿Conclusions

We hope to have demonstrated that Sharkey et al. (2021a) did not equate BINs with species. This is exemplified by several cases in which multiple species were uncovered in single BINs. We reiterate that BINs were used as a preliminary sorting tool, just as a morphological approach might preliminarily sort specimens by color, size, or other traits. The fact that the match between BINs and species concepts was well over 90% does not equate to equality. We explain that consensus barcodes were used only to provide a diagnosis that conformed to the rules of the International Code of Zoological Nomenclature and agree with Meier et al. (2021) that they do not provide a functional diagnosis other than what is similarly provided by consensus morphology. We emphasize that minimalist revisions will facilitate future taxonomic research, not hinder it. We refute the claim that the BOLD sequences of *P.vanachterbergi* are pseudogenes and demonstrate that they simply represent a frameshift mutation. We reassert our claim that morphological evidence alone is insufficient to delineate many species, but its usefulness lies in traits that are congruent with molecular data. Finally, we show that in the cases in which COI barcodes code for the same amino acids, morphological and host use data reaffirm their utility as do the barcodes themselves in their various combinations of nucleotides.
